# Physiology, Nutrition, and Postharvest Technology on Shallots (*Allium cepa* L. *aggregatum*): A Review

**DOI:** 10.1155/sci5/8328535

**Published:** 2026-05-31

**Authors:** Sulusi Prabawati, Siti Mariana Widayanti, Anna Sulistyaningrum, Setyadjit Setyadjit, Abdullah Bin Arif, Christina Winarti, Irpan Badrul Jamal, S. Joni Munarso, Waryat Waryat, Agus Budiyanto, Risfaheri Risfaheri

**Affiliations:** ^1^ Research Center for Horticulture, National Research and Innovation Agency, Jakarta, Indonesia, brin.go.id; ^2^ Research Center for Food Technology and Processing, National Research and Innovation Agency, Jakarta, Indonesia, brin.go.id; ^3^ Research Center for Process Technology, National Research and Innovation Agency, Jakarta, Indonesia, brin.go.id; ^4^ Research Center for Equipment Manufacturing Technology, National Research and Innovation Agency, Jakarta, Indonesia, brin.go.id

**Keywords:** bulb crops, curing, drying, fresh handling, quality, storage

## Abstract

Shallots (*Allium cepa* L. *aggregatum*) are an important horticultural commodity with high economic value and a strategic role in food security and the culinary industry, particularly in Southeast and South Asia. Shallots are a key ingredient in many dishes and are valued for their bioactive compounds, including flavonoids such as quercetin, organosulfur compounds, and natural antioxidants. These compounds contribute to various health benefits, including antimicrobial, anti‐inflammatory, and anticancer effects. Shallots are classified as nonclimacteric plants, meaning they do not continue to ripen after harvest. Tropical shallot varieties grown in lowland regions typically reach maturity around 60 days after planting, characterized by stems that are 60%–80% weakened. However, after harvest, physiological changes such as respiration and transpiration can reduce their quality. Because shallots are highly perishable particularly during peak production seasons when storage capacity is limited, effective postharvest handling practices such as curing, drying, and proper storage are essential. These methods help prevent decay, reduce losses, and extend the shelf life and availability of fresh shallots.

## 1. Introduction

Shallots (*Allium cepa* L. var. *aggregatum*) and common onions (*Allium cepa* L. var. *cepa*) belong to the same species but differ significantly in their botanical characteristics, morphology, and chemical composition. Understanding these distinctions is essential for researchers, breeders, and producers seeking to optimize cultivation practices and postharvest handling techniques. Shallots, known as a multiplying or clustering variety, form several small bulbs (cloves) that grow in compact clusters from a single planted bulb, while common onions typically produce a single large bulb. Morphologically, shallots exhibit a smaller, elongated shape with reddish‐purple papery skins, whereas onions are generally larger, rounder, and available in white, yellow, or red varieties. Chemically, shallots contain higher levels of flavonoids especially quercetin and organosulfur compounds that contribute to their antioxidant, anti‐inflammatory, antimicrobial, and anticancer properties [1, 2].

Agronomically, shallots (*Allium cepa* L. var. *ascalonicum* or *aggregatum*) differ from onions and other *Allium* species in several key aspects. The most fundamental distinction lies in bulb formation: onion plants usually produce a single bulb, while shallots form between three to 10 bulbs per clump [3, 4]. Shallot bulbs are smaller, more elongated, and have reddish purple skins with denser, drier, and thinner scales that emit a sharper aroma. In contrast, onions have thicker, juicier layers with a milder scent and can display yellowish brown, white, or reddish purple outer skins. Shallots also have a shorter growth cycle, reaching maturity in 55–70 days after planting when the foliage wilts and the stem neck softens, whereas onions require about 90–120 days to mature [5]. Additionally, shallots adapt well to tropical climates such as Indonesia, thriving in lowland to mid‐altitude areas (0–800 m a.s.l.) with full sunlight exposure, while onions perform best in cooler, highland regions above 800 m [3, 6]. However, in countries with four distinct seasons, shallot growth patterns differ from those observed in Indonesia, likely due to ecological differences.

Beyond their agronomic value, shallots are recognized as medicinal and functional plants with substantial potential in traditional and modern medicine. They are widely used as culinary spices, food industry ingredients, and therapeutic agents [7, 8]. Phytochemical studies have identified polyphenolic compounds such as catechin, quercetin, apigenin, ajoene, gallic acid, sapogenin, thiosulfate, and kaempferol in shallots [9, 10]. Other bioactive components include sugars, amino acids, vitamins, minerals, sulfur compounds, enzymes, phytohormones, flavonoids, and saponins [7, 10]. Notably, organosulfur compounds like diallyl disulfide, diallyl trisulfide, and allyl propyl disulfide contribute to their strong antioxidant capacity and distinctive aroma. Essential oils, making up approximately 61.14% of the total composition, further enhance their functional and medicinal properties. Such attributes position shallots as promising candidates for nutraceutical and pharmaceutical applications [11, 12].

Despite their economic and medicinal importance, shallots are highly perishable due to their high moisture content exceeding 80%. They are susceptible to postharvest deterioration, including bulb softening, wrinkling, sprouting, decay, and fungal infestation. Postharvest losses can reach 20%–40%, largely caused by inadequate curing and drying processes. Improper handling and storage not only accelerate spoilage but also degrade the bioactive compounds responsible for their therapeutic effects. Thus, effective postharvest management is crucial to maintaining quality and extending shelf life. This paper aims to review the physiology, nutritional composition, and postharvest handling technologies of shallots, highlighting the need for improved preservation strategies to reduce losses and enhance the market potential of this valuable yet delicate crop.

## 2. Harvest Maturity

Handling of shallots begins from harvest, determining the maturity of the shallot bulbs. Farmers are accustomed to determining the maturity of the shallot bulb harvest based on its age since planting, as indicated by the weakening of the stem neck, the plant’s appearance resembling a lying down posture, and the leaves turning yellow. Table 1 shows the harvest maturity of several shallot varieties. The popular tropical shallot variety is Bima, which is planted in the lowlands, has a harvest age of 60 days, and is characterized by 60% of the stems weakening. Ismail [23] stated that the Bima variety of shallots can be harvested 50–60 days after planting, as indicated by the consistent characteristics of the bulbs (weight, diameter, water content, and TSS), which remain unchanged.

**TABLE 1:** Harvest maturity of several varieties of shallots.

No.	Variety	Organ for planting	Location altitude	Rainfall	Harvest maturity (day)	Harvest criteria	Reference
1	Bima	Bulb	—	—	60	60% stem weakens	[13]

2	Batu Ijo	Bulb	Lowland		55–60	80% stem weakens	[14]

3	SS Sakato	Bulb	1458 – 1500 m above sea level	212 days/year	85–95	80% stem weakens	[15]

4	Huruta	Bulb	1340–2200 m above sea level	700–1386 mm	103.61	—	[16]
	Negelle				103.11	—	

5	Minjar	Bulb	1600–2200 m above sea level	> 700 mm	101	—	[17]
	Negelle		600–800 m above sea level		95–120	—	
	Huruta		1800–2200 m above sea level				

6	Batu Ijo	Bulb	Toba Samosir district (highland)	—	60	—	[18]

7	Vethalan	Bulb	1900 m above sea level	—	110–125	—	[19]
	Minjar						

8	DZSHT‐91‐2B (Debre Zeit)	TSS	1900 m above sea level	—	124–155	—	[19–21]
	DZSHT‐193‐1A				120–155		
	DZSHT‐157‐1B				105–140		

9	Trisula	TSS	1150–1250 m above sea level	—	130–147		[22]

10	Vethalan	True shallot seed (TSS)	1900 m above sea level	—	113–150	—	[19]
	Minjar						

Differences in harvest maturity among various shallot cultivars and accessions have also been studied by [24]. Varieties and temperatures affect the harvest age of shallot bulbs. The Batu Ijo variety is harvested at 55–60 days in the lowlands, while in the highlands with cooler temperatures, it is harvested at 65–70 days. The genetic properties of the variety likely cause differences in harvest maturity between varieties. Yeshiwas et al.’s [17] research, which tested four varieties with different planting altitude locations, showed that the four varieties reached different maximum physiological maturity levels, and shallots planted at higher altitude locations reached maximum physiological maturity later. Differences in varieties and planting locations significantly affect physiological maturity in shallots, which is caused by the genotype of the variety and the temperature of the planting location [17]. However, another study reports that its evaluation found that varieties do not affect shallot bulbs in reaching 75% physiological maturity, regardless of whether they are bulb seeds or seed varieties [25].

The harvest maturity of the bulb also affects the chemical content of shallots. Bulb harvest at 75% top fall has higher bulb production and the highest dry matter content. The pyruvate content, reflecting pungency, reaches a maximum, and the soluble solid content falls to 100%. When viewed from the perspective of root damage, the best option is a 100% top fall harvest [26]. This means that the harvest at 75% top fall of the bulb is already optimum, with high nutritional quality. This condition is related to the perfect filling of the bulb from photosynthesis.

Tabor [19] has developed shallots using shallot seeds, as planting seeds from bulbs has several disadvantages, including the need for significant storage space and low productivity. In addition, bulbs are more susceptible to fungi such as *Fusarium* spp and latent viruses carried from previous generations [22]. So, using shallot seeds is expected to be a practical breakthrough in increasing crop yields, productivity, and space efficiency and is easy to distribute. Based on Table 1, shallot cultivation using bulbs generally results in a shorter harvest period, ranging from 60 to 85 days. In contrast, cultivation using seeds (true shallot seed or TSS) requires approximately 120–155 days. According to Saidah et al. [27], the harvest period for shallots grown from TSS is 19–26 days longer compared to those grown from bulbs. This is primarily due to the nursery phase required for TSS, which takes approximately 30 days. In addition, bulbs contain energy reserves and active shoots, allowing them to grow and form bulbs more rapidly. However, shallots grown from TSS produce significantly higher bulb weights, up to twice as much, with larger bulb sizes compared to those grown from traditional bulb seeds.

In planting conditions with the same height, planting with seeds has a longer harvest age compared to planting with bulbs. However, it has several advantages, including higher yields, uniform bulbs, and disease resistance. Differences in location have a significant impact on the maturity level of the bulb harvest. The Debre Zeit variety has a shorter harvesting maturity when planted on Alfisol soil (124–139 days), while in Vertisol soil conditions, the harvest age is 155 days. This is because Vertisol soil conditions result in high water retention capacity and increased plant vigor. However, plants with a more extended harvest period will produce better yields. In Alfisol soil conditions, seed varieties such as Debre Zeit have a productivity of around 42.73–54.91 t/ha, while the Vethalan variety yields 51.90 t/ha, and for the Minjar variety, it is 21.95 t/ha [19]. Although the harvest period is longer, it yields almost twice as much. According to research results from Rosliani et al. [22], TSS is considered much more profitable than seed bulbs. To plant shallots on 1 ha of land, around 1.2–1.5 tons of bulbs are needed as seeds, while TSS only requires 3–5 kg. This results in a lower cost of seed production. According to Hilman et al. [28], TSS has longer storage viability compared to bulb‐derived seed. Bulb seed can only be stored in warehouses for a maximum of 4 months, whereas TSS can maintain up to 50% germination capacity even after 1–2 years of storage. However, the production of TSS faces challenges due to low flowering and seed formation rates. Besides this, TSS requires highland conditions with temperatures ranging from 16°C to 18°C.

## 3. Postharvest Physiology and Quality Changes of Shallots During Storage

In Indonesia, shallots are commonly stored using traditional methods at ambient temperatures of 25°C–30°C and relative humidity (RH) of 70%–80%, which typically results in a weight loss of approximately 25%. Environmental control during storage, particularly regulation of temperature and humidity, has been shown to reduce weight loss by about 10%–17% [29]. Low temperature storage can slow down metabolic processes, thereby extending the shelf life of shallots [30]. Additionally, maintaining low temperatures can inhibit the growth of undesirable microorganisms. A study conducted by Nurkomar et al. [31] investigated shallot storage at temperatures of 10°C and 20°C with RH 65%–75% and at room temperature with uncontrolled RH. The results indicated that storage at 10°C with RH 65%–75% was the most effective treatment, as it successfully reduced the respiration rate of shallots.

Preharvest factors affect the quality of onions during storage. Woldetsadik and Workneh [26] stated that the storage of shallot bulbs after harvesting poses a problem for growers in tropical regions due to postharvest losses, including reduced bulb weight, bulb rot, sprouting, and rooting. Moreover, all these problems lead to shallot unmarketability caused by increased N application. According to Kale [32], a complex interaction of pre‐ and postharvest factors, which include mineral nutrition, cultivar, bulb maturity, and conditions during maturation, harvesting, and curing, affects bulb shelf life. Getahun et al. [33] observed that the time of harvesting and the season in which the shallot was stored had a significant influence on the quality of the shallot bulb and yield after storage. Hussen [34] notes that the lower moisture content of the bulbs gradually increased the dry matter content of the onions, regardless of the duration of drying or curing.

According to Katherine et al. [35], a slight increase in dry matter occurs due to the loss of moisture from the outer surface. In contrast, the subsequent reduction corresponds to the hydrolysis of fructans and the termination of the dormancy period, during which the bulbs begin to sprout. The respiration pattern of shallots during storage is strongly influenced by temperature and water content. Rapid respiration accelerates water loss, leading to decreased bulb weight and reduced freshness. Shallots stored at high temperatures and low RH tend to undergo faster respiration, resulting in greater water loss and shorter shelf life. In high temperature, other chemical and biochemical reactions occurred. Conversely, shallots with high initial water content are more susceptible to damage and spoilage. Storage at low temperatures can suppress the respiration rate, thereby extending the storage life of shallots. The optimal storage conditions for maintaining shallot quality are temperatures between 7°C and 10°C with a relative humidity of 65%–75%.

### 3.1. Physical Characteristics

**FIGURE 1:**
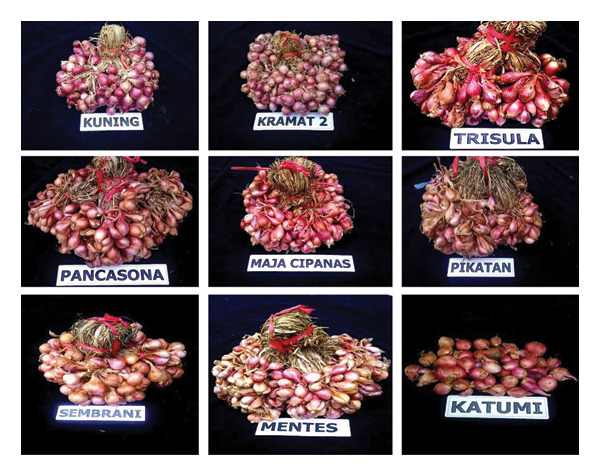
The varieties of shallots in Indonesia [37].

**FIGURE 2:**
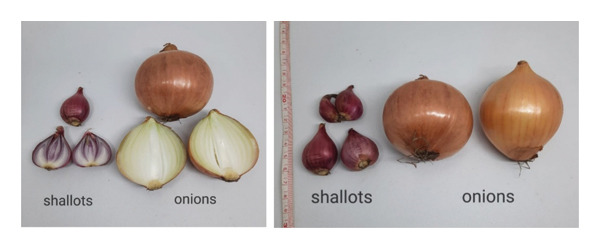
Physical and size differences between shallot and onion bulbs.

Shallots are a common spice in Indonesian households, valued for their unique physical, chemical, and functional properties. Besides being used as a flavoring ingredient in various dishes, shallots also offer health benefits and serve as a preservative. Understanding their physical characteristics is crucial in determining whether they are suitable for fresh consumption or processing. Consumers generally prefer large shallots (8–10 g per bulb) with shiny red skin [36, 37]. The main physical traits of shallots include bulb weight, size, number, and color. According to Sukasih et al. [37], 10 varieties of shallots such as Pancasona, Kuning, Katumi, and others show bulb diameters ranging from 1.35 to 3.00 cm, bulb weights of 2–12 g, and 1 to 3 bulbs per cluster (Table 2 and Figure 1). These findings support Azmi et al. [36], who reported bulb diameters of 1.7–2.4 cm and weights of 5.3–6.7 g. The color of shallot bulbs varies from pale red to dark red, attributed to the presence of anthocyanin pigments. Comparisons with other cultivated varieties, such as Batu Ijo, show that it has a higher bulb weight; as reported by Rachmawati et al. [38], the weight of the bulb is 15–20 g/bulb. Figure 2 shows the size differences between shallots and onions.

**TABLE 2:** Physical characteristics of various shallots.

No.	Variety	Diameter of bulbs (cm)	Length of bulbs (cm)	Weight of bulbs (g)	Diameter of bulbs (cm)	Length of bulbs (cm)	Weight of bulbs (g)	Number of bulbs	Skin color
		Main bulbs			Tiller bulbs				
1	Pikatan	1.60 ± 0.2	2.50 ± 0.1	2.68 ± 0.2	1.12 ± 0.1	1.87 ± 0.1	1.72 ± 0.1	3.00 ± 1	Pale red
2	Pancasona	1.80 ± 0.1	2.45 ± 0.1	3.55 ± 0.1	1.28 ± 0.1	1.96 ± 0.2	1.92 ± 0.1	3.00 ± 1	Red
3	Katumi	1.85 ± 0.1	2.27 ± 0.1	4.16 ± 0.2	0.80 ± 0.1	1.15 ± 0.1	0.41 ± 0.2	1.00 ± 0.5	Red
4	Trisula	1.75 ± 0.1	2.95 ± 0.1	4.22 ± 0.1	1.53 ± 0.1	3.03 ± 0.1	2.84 ± 0.1	2.00 ± 0.5	Red
5	Majalok Lembang	1.90 ± 0.1	2.73 ± 0.2	2.73 ± 0.2	1.20 ± 0.2	2.00 ± 0.1	1.43 ± 0.1	1.00 ± 0.5	Red
6	Kuning	2.02 ± 0.1	2.35 ± 0.2	6.04 ± 0.1	1.60 ± 0.1	2.10 ± 0.1	3.22 ± 0.1	2.00 ± 1	Dark red
7	Kramat‐2	1.82 ± 0.1	2.54 ± 0.1	4.11 ± 0.1	1.26 ± 0.1	2.02 ± 0.1	1.92 ± 0.1	2.00 ± 0.5	Red
8	Mentes	1.35 ± 0.1	2.27 ± 0.1	2.35 ± 0.1	1.08 ± 0.1	1.94 ± 0.1	1.24 ± 0.1	3.00 ± 0.5	Pale red
9	Sembrani	3.08 ± 0.2	2.85 ± 0.1	12.59 ± 0.1	2.30 ± 0.1	2.50 ± 0.1	5.30 ± 0.2	2.00 ± 0.5	Yellowish
10	Bima Lokal	1.82 ± 0.1	3.02 ± 0.1	4.33 ± 0.1	1.57 ± 0.2	2.70 ± 0.1	2.63 ± 0.1	2.00 ± 0.5	Red

*Note:* Source Sukasih et al. [37].

### 3.2. Chemical Characteristics

The variety, growing environment, postharvest handling, curing, and storage influence the chemical composition of shallot bulbs. The nutritional composition and phenolic content, as well as the mineral content, of the shallot bulbs, shallot flour, and onions are presented in Tables 3 and 4, respectively. The chemical composition of shallot bulbs is higher than that of onions. Based on the nutritional composition and bioactive components contained in shallots, this bulb offers various benefits, including serving as a cooking spice and a raw material for the food and pharmaceutical industries [7, 8]. In addition to carbohydrates, shallots are used as a source of minerals and vitamins, as well as other bioactive components (gallic acid, apigenin, eriodictyol, quercetin, isoquercetin, kaempferol, catechin, and tannin). Shallots contain antioxidant components such as diallyl disulfide, diallyl trisulfide, and allyl propyl disulfide [7]. In addition, shallots are rich in essential oils, with a high content of organosulfur compounds at 61.14%, which is an important bioactive component [18]. This compound is the hallmark of the shallot flavor.

**TABLE 3:** Nutritional composition, quercetin levels, and phenolic content of shallot bulbs and onions.

Parameters	Shallot bulbs	Onions
Moisture (g/100 g)	80.03–87.51	87.00–89.00
Ash (g/100 g)	0.55–0.80	0.9–1.1
Fat (g/100 g)	0.03–0.59	0.7–1.3
Protein (g/100 g)	2.36–4.23	1.5–3.0
Carbohydrate (g/100 g)	8.08–16.33	6.00–10.00
Fiber (g/100 g)	1.90–2.61	0.6–1.00
Starch (g/100 g)	3.44–4.73	0–1.0
Quercetin (ppm)	218.91–1766.40	100–300

References	[37, 39]	[40]

**TABLE 4:** Mineral content of shallot bulbs.

Parameters	Shallot bulbs	Shallot flour
Ca (mg/100 g)	37	216
Mg (mg/100 g)	21	0.71
K (mg/100 g)	334	15.8
Na (mg/100 g)	NA	14.7
Fe (mg/100 g)	1.2	2.96
Zn (mg/100 g)	0.4	NA
Mn (mg/100 g)	0.292	1.09
P (mg/100 g)	60	3.18
S (mg/100 g)	NA	2.18

References	[39, 41]	[42]

In addition, the content of antioxidant compounds is also relatively high, including sulfur compound derivatives (diallyl disulfide and diallyl trisulfide), flavonoids (glucosides of quercetin), polyphenols, ascorbic acid, and saponins [43]. For this reason, shallots also have great potential as functional food ingredients because of their antioxidant and bioactive compounds.

Quercetin is a flavonoid compound abundantly present in shallots (*Allium ascalonicum*), known for its diverse biological activities. It possesses significant anti‐inflammatory, antihistaminic, antiallergic, anticancer, and antiviral properties [44]. The concentration of quercetin in shallot bulbs ranges from 218.91 to 1766.40 ppm, depending on factors such as cultivar type, environmental growing conditions, and the extraction technique used. Quercetin exists in both aglycone and glucoside forms, with the latter demonstrating superior absorption efficiency compared to nonglucosidic forms. Interestingly, the bioavailability of quercetin‐3‐glucoside and quercetin‐4′‐glucoside is reported to be similar, with no significant difference between these two glycosidic variants [45].

Shallots contain essential nutrients, including carbohydrates, protein, vitamins (C, B1, B2, B3, B6, B9, E, K, and A), and minerals such as Ca, Fe, Mg, P, K, Na, and Zn [39]. They are particularly rich in potassium, which plays a vital role in metabolism, maintaining blood pressure balance, preventing arterial hardening, clearing bad cholesterol from blood vessels, and regulating muscle contractions and nerve and brain functions. The calcium and phosphorus in shallots contribute to healthy bones and teeth. The amino acid composition of six shallot cultivars studied by [46] included L‐serine, L‐glutamic acid, L‐phenylalanine, L‐isoleucine, L‐valine, L‐alanine, L‐arginine, glycine, L‐lysine, L‐aspartic acid, L‐leucine, L‐tyrosine, L‐proline, L‐threonine, L‐histidine, L‐cystine, and L‐methionine. Consistent with Indonesian shallot characteristics, glutamic acid is found in high concentrations along with asparagine, serine, histidine, threonine, proline, and valine [47].

### 3.3. Postharvest Diseases

Postharvest diseases can significantly damage shallot bulbs during storage. Fungi are the main microorganisms responsible for these diseases (Table 5), while bacterial infections, such as *Erwinia carotovora* (the causative agent of soft rot), can exacerbate the damage. This condition is similar to that of onions, where bulb rot is primarily caused by fungi [57]. Moreover, certain postharvest diseases are linked to microbial infections that begin while the shallots are still in the field. Several fungal species from the genus *Fusarium* are known to cause pre‐ and postharvest infections [52, 58–60]. These fungi cause leaf twisting disease and bulb rot, resulting in yield loss. Resistance varies among varieties, with larger bulbs that have thicker layers showing greater resistance [61].

**TABLE 5:** Postharvest diseases of shallot bulbs during storage.

Microorganism group	Disease/rot	Symptom	Disease/rot‐causing organism	Reference
Fungi	Basal rot	Advanced attack, tuber tissue becomes soft and watery, the basal part is dark brown	*Fusarium*	[48]
	Basal rot	Not described	*F. oxysporum* f. sp. *cepae*, *F. solani*	[49]
			*F. proliferatum*	
	Black rot	The rotten parts of the tubers contain black spores	*Aspergillus niger*	[50]
	Basal rot	Rot starts at the base of the bulb	*Fusarium oxysporum*	
	Anthracnose	Sunken spots on the surface of the bulb	*Colletotrichum gloeosporioides*	
	Basal rot	Initially the fungal mass is white, the rotting bulbs are brownish or blackish	*Fusarium*	[51]
		Pigmentation, discoloration, and decay	*Aspergillus*	
			*Mucor*	
			*Sclerotinia*	
	Anthracnose	The surface of the bub has dry, sunken wounds	*Colletotrichum gloeosporioides*	[52]
			*Alternaria alternata*, *Aspergillus niger*	
			*Fusarium fujikuroi*	
			*F. oxysporum*	
			*F. solani*	
			*Penicillium citrinum*	
			*P. pinophilum*	
	Basal rot		*Fusarium oxysporum* f. sp. *cepae* (Snyder and Hans)	[20, 21]

Bacteria	Soft rot	The bulbs become soft and rotten	*Erwinia carotovora*	[50]
	Bulb rot	The bulbs become rotten	*Pseudomonas aeruginosa*	[53]
	Bulb rot	Discoloration of inner bulb to brown or blackish	*Enterobacter cloacae*	

Virus		Viral infection symptoms are not observed in the bulbs but become evident on the leaves during field growth	*Shallot yellow stripe virus* (SYSV)	[54]
			GCLV (*Garlic common latent virus)*	[55]
			SLV (*Shallot latent virus)*	[56]
			Potyvirus	
			SLV *(Shallot latent virus)*	
			GCLV *(Garlic common latent virus)*	
			SYSV *(Shallot yellow stripe virus)*	
			OYDV *(Onion yellow dwarf virus)*	

*Fusarium oxysporum* is the main pathogen responsible for basal rot in various types of onions and shallots, producing symptoms such as browning and white mycelium at the stem base [62]. Infections can occur as early as the seed germination stage but usually become visible only after harvest [60]. Other species, including *F. verticillioides*, *F. solani*, and *F. proliferatum*, are also known to cause similar damage [58]. The progression of *Fusarium* infection is accelerated under high temperatures (25°C–32°C) [48, 62]. Recent studies emphasize environmentally friendly approaches to manage basal rot rather than chemical fungicides. According to Wesoly et al. [63], infected shallots exhibit elevated levels of volatile compounds, such as methyl propyl sulfide and dimethyl disulfide, which can serve as early biochemical markers of basal rot. Furthermore, Herlina et al. [64] found that *Fusarium*‐infected shallots show higher secondary metabolite diversity and expression, especially in the susceptible Katumi variety, indicating potential for biological management of *Fusarium*‐related diseases.

Other fungi such as *Aspergillus niger*, *Mucor*, and *Sclerotinia* also contribute to rot in shallots [51, 52, 59, 65]. *A. niger* infection initially appears as black spots on bulb surfaces, developing into black mold under high humidity [66]. This fungus spreads rapidly through air, water, and soil, though its growth can be suppressed in dry, low‐humidity storage environments. In addition to shallots, *A. niger* also affects onions, grapes, peanuts, and other vegetables, leading to significant postharvest losses and serving as a major food contaminant [67, 68]. Dharmaputra et al. [52] reported that shallot bulbs of the Bima variety are susceptible to anthracnose caused by the fungus *Colletotrichum gloeosporioides*, which is noted for its high pathogenicity. Similarly, Thu et al. [50] identified *C. gloeosporioides* in shallots, however it was not the predominant pathogen in their findings. Other studies, including those by [69, 70] have also highlighted *C. gloeosporioides* as a significant cause of anthracnose in shallot plants.

Reports on postharvest losses in shallots due to bacterial infection remain limited. However, Thu et al. [50] reported infections caused by *Erwinia carotovora* and *Pseudomonas aeruginosa*, with *E. carotovora* identified as a major pathogen. *Enterobacter cloacae* was found infecting shallot bulbs in Indonesia, producing symptoms 8–11 days after inoculation and leading to brown to black rot in advanced stages [53]. In addition, viral infections have also been detected in shallot bulbs, including *Shallot yellow stripe virus* identified using RT‐PCR [54]. Kadwati and Hidayat [55] reported that shallot bulbs were infected by *Garlic common latent virus*, *Shallot latent virus*, and a potyvirus using ELISA assays. These viruses may occur as single or mixed infections, and more recent studies confirmed the presence of OYDV, SYSV, SLV, and GCLV [56]. Although viral symptoms typically appear after planting, infected bulbs act as primary inoculum, causing leaf symptoms such as yellow mosaic, stripes, chlorotic spots, and wrinkling, ultimately reducing yield.

Microbial infections result in both physical and chemical deterioration of shallot bulbs. While specific data for shallots are limited, studies on onions reveal that *A. niger* infection increases water content from 86.80% to 89.42% and reduces crude fiber, ash, ascorbic acid, protein, and fat contents [71]. This nutrient degradation indicates significant biochemical changes caused by fungal activity. Among all pathogens, *Fusarium* is the most destructive, as infection can occur pre‐ or postharvest, leading to soft, watery bulbs due to internal tissue decay. Moreover, high storage temperatures of 28°C–32°C further accelerate basal rot development and facilitate its spread to other bulbs, underscoring the need for proper temperature and humidity control during shallot storage.

## 4. Postharvest Handling of Fresh Shallots

The postharvest handling of shallots consists of several stages, including curing, shallot bulb drying, and shallot storage. The shallot storage comprises several activities, namely, drying, smoking, low‐temperature storage, modified atmosphere storage, and controlled atmosphere storage (CAS). This series of processes is carried out to maintain the quality of shallot bulbs in terms of physical, chemical, bioactive , and microbiological aspects, so that shallot bulbs have a longer shelf life with good quality.

### 4.1. Conventional and Artificial Curing and Drying Processes for Shallots

#### 4.1.1. Curing

Curing is a crucial postharvest process that has a direct impact on the physical, chemical, and biochemical properties of bulb crops. The curing process is the first postharvest handling process carried out after the shallots are harvested. Harvesting is carried out depending on the variety planted. The curing process is intended to reduce the water content around the leaves and neck of the bulb. During this curing process, an epidermis layer will form that covers the injured part of the bulb skin due to scratches during harvesting.

The conventional curing process is carried out by utilizing sunlight. Therefore, the duration of the curing process is highly dependent on the intensity of the sun (Table 6). The most significant energy in this curing process is used to wilt the leaves. Therefore, a study conducted by [74] on the treatment of cut spring onions, specifically those about 1–10 cm and 11–20 cm from the base of the leaf, found that this can accelerate the curing and drying processes. Faster curing and drying processes can reduce the risk of bulbs being attacked by rotting bacteria.

**TABLE 6:** Technology for the curing process of shallot bulbs.

Treatment	Temperature/storage duration	Quality changes	Reference
Sun drying	3–5 days	Weight loss (3%–3.5%)	[72]
Cabinet dryer	45°C ± 0.6°C and RH 69%, 92 h	Bulb moisture of 79.92%, skin bulb moisture of 56.8%, VRS 33.65, and SSC of 19.44%	[73]
Leaf cutting	27°C–30°C	Decreased weight loss; the color, moisture content, and texture tend to be constant	[74]
In‐store drying (ID)	Temperature of 40°C–45°C and RH of 70%–75% during 48 h	Damage to shallot bulbs can be suppressed during 8 weeks of storage with a lower weight loss of 10.58%	[75]
ID with sun drying	—	Faster curing, reducing yield loss by 11%; shallot bulb quality and quantity can be maintained (color, odor, taste, and nutrition)	[76]

Harvesting shallots during the rainy season requires a longer curing process than harvesting shallots during the dry season. Therefore, during the rainy season, the risk of shallots being exposed to microorganisms/bacteria that cause rot and loss of harvest will be higher. Farmers in the Brebes area typically carry out the curing process using sunlight for 3–5 days, depending on the weather conditions. The curing process also aims to reduce dirt in the form of soil that is carried during harvest. Incomplete curing will cause the onion bulbs to rot quickly, as the relatively high water content at the base of the bulb provides an ideal environment for the development of base rot bacteria. This often occurs when harvesting onions in the rainy season. Weight loss from harvest to curing is 3%–3.5% [72]. The weight loss that occurs during the curing process actually comes from the soil that sticks to the onion bulbs during harvest and falls off when the soil dries. Weight loss due to respiration and transpiration during curing is minimal. The water content of shallots at harvest time is approximately 88%–90%. After the curing and drying processes, the water content decreases to around 86% [75].

Modification of the curing process of shallots has been investigated by [73], who conducted the curing of harvested shallots using a cabinet dryer set at a temperature of 45°C ± 0.6°C and relative humidity (RH) of 69% ± 0.4% for various durations: 0, 32, 80, 92, and 104 h. The results indicated that curing at 45°C produced high‐quality shallots with improved physical characteristics, including a perfectly dried outer skin and neck, increased bulb diameter, greater hardness, enhanced red color intensity, and higher levels of total dissolved solids and volatile reducing substances (VRS).

This curing process plays a crucial role, as it significantly impacts the overall quality of the shallots. During drying, the water content in the shallot bulbs decreases rapidly at the beginning of the process and then gradually slows down over time. After approximately 80 h, the rate of evaporation declined due to the formation of the outer skin layer, which acts as a barrier to moisture loss.

The adequacy of the curing process can be identified through several indicators, such as the narrowing of the bulb neck and the production of a dry, rustling sound when the outer skins are rubbed together. Among the treatments tested, the 92‐h curing duration resulted in the best quality bulbs, characterized by a water content of 79.92% in the tubers, 56.8% in the outer skin, a VRS value of 33.65, and a total dissolved solids content of 19.44%.

The formation of a dry outer layer on the bulbs serves as a protective barrier against mechanical, physical, and microbiological damage. Therefore, an optimal curing duration of approximately 92 h at 45°C not only enhances the physical appearance and chemical composition of shallots but also extends their shelf life by minimizing the potential for postharvest deterioration.

#### 4.1.2. Drying

The drying process is carried out after the curing process. The purpose of drying shallots is to maintain the moisture content in the bulbs at a level close to that of fresh conditions, so that the water content of the bulbs remains almost unchanged compared to when they are fresh. Therefore, drying is only intended to reduce the moisture content in the spring shallots and shallot skins by 1–2 layers of the bulb skin. Conventional drying generally lasts for 7–10 days [77], depending on the weather so if the shallot harvest occurs during the rainy season, it will usually cause relatively high damage due to the still high moisture content, which will also affect the color, smell, taste, and nutrition and the shelf life of the shallots becomes shorter. Several studies have been conducted to investigate the optimal drying technology for maintaining the freshness of shallot bulbs during storage (Table 7).

**TABLE 7:** Drying technology to maintain the freshness of shallot bulbs.

No.	Drying method	Effect	Reference
1.	Direct sun drying	Reduce yield loss by 11% and maintain bulb quality	[76]
2.	Direct sun drying with bamboo woven base	Reduce yield loss by 2.08% up to 3 months of storage period	[78]
3.	Instore drying	Losses can be suppressed during 8 weeks of storage around 10.58%	[75]

In conventional drying, farmers typically dry shallots directly on the ground without a base, making them vulnerable to microbial contamination from the soil. Lestari et al. [78] found that using bamboo nets, either coated with plastic or not, reduces direct soil contact, minimizes dirt contamination, and allows more even heating due to better air circulation. This method resulted in a 2.08% lower yield loss and slightly higher moisture content (84.31%) compared to the conventional method (83.08%). However, most farmers still rely on sunlight for drying, which becomes problematic during the rainy season. To overcome weather‐related limitations, mechanical drying methods have been developed, such as blowing hot air at 46°C for 10 h with 70%–80% humidity or drying at 30°C–35°C with RH > 75% or 40°C–45°C with RH 70%–75% for 48 h [75]. These mechanical methods effectively reduce weight loss and bulb damage during up to 8 weeks of storage.

The use of ID as a postharvest handling solution for shallots is one approach to overcoming postharvest problems in this crop. Using ID can reduce the water content in the outer skin of the shallot. By drying the neck (the tip of the bulb or base of the leaves), the bulb does not experience a significant decrease in weight, and openings for microbe infestation are no longer available. This process maintains the color and texture of the shallots, dries them faster by 3 days compared to conventional methods, avoids pests and diseases because it is indoors, and can reduce the level of damage by 11% compared to conventional drying. Histifarina et al. [76] conducted a study comparing the drying of shallots using two conventional methods and ID to assess the quality of shallots. ID, with a capacity of 5–10 tons, features a zinc roof and is equipped with air aeration, along with walls made of fiberglass. Financially, drying using ID is more efficient (58.26%) when compared to conventional drying and is financially feasible (net B/C 1.85, R/C 1.27) and has a PBP of 4.8 months, which means that this technology will be profitable (return the investment capital that has been issued) after 4.8 months of use. If we examine the physical characteristics, the use of ID produces better quality shallot bulbs, specifically with a weight loss of 29.24%, an average bulb weight of 7.01 g, and a hardness of 1.93 mm/s per 100 g. When compared to conventional methods, it can reduce weight loss by 5.94%. The chemical content of total dissolved solids is higher, reaching 17.43%.

In addition to drying technology, appropriate storage technology is also important to maintain the freshness and quality of shallot bulbs during storage (Table 8). Low‐temperature storage at 0°C with RH 65%–70% was reported to reduce weight loss and maintain the quality of Bima Brebes, Tajuk, and Bali Karet shallot varieties [79]. Storage at 5°C can also maintain shallot quality for up to 8 weeks with low weight loss (7.06%) and stable VRS content (26.53 µEq/g) [29]. Other storage technologies, such as ID with good air circulation, can maintain the quality of shallot bulbs with lower weight loss. The use of smoking as a natural antimicrobial can also reduce storage room humidity and suppress bulb deterioration while producing a distinctive aroma. In addition, the use of CAS with low oxygen concentration and higher carbon dioxide concentration was reported to be effective in maintaining dry weight, firmness, total soluble solids, germination, fructan content, and pyruvate content during long‐term storage [80, 81].

**TABLE 8:** Storage technology to maintain the freshness of shallot bulbs.

No.	Existing	Technology	Result	Reference
1	Room temperature 25°C–32°C, RH 50%–88%	Low temperature storage: temperatures 0°C and 5°C, RH 65%–70% for 3 months	Storing at 0°C resulted in the best tuber quality with the lowest weight loss for all three Bima Brebes, Tajuk, and Bali Karet varieties	[79]
2		Storage temperature 5°C	Shallots with an initial moisture content of 80% stored at 5°C maintain the best quality for up to 8 weeks of storage, with a weight loss of 7.06% and a VRS of 26.53 µEq/g	[29]
3		Storage with an in‐store dryer (room temperature with good air circulation)	Lower weight loss, the quality parameters of fresh shallots can be maintained for longer	[76]
		Smoking (as a natural antimicrobial)	Reduces storage humidity, thereby decreasing tuber spoilage and the formation of a distinctive aroma	
		CAS (5% CO_2_+5% O_2_)	7 months material weight, dry weight, hardness, and dissolved solids content	[80]
			Using 3.03 kPa CO_2_ and 5.05 kPa O_2_ at 2°C for 80 days can maintain dry weight, hardness, germination, total dissolved solids, fructan concentration, and pyruvate concentration	[81]

## 5. Postharvest Technology of Shallots During Storage

### 5.1. Cold Storage

Cold storage of shallot bulbs aims to reduce the respiration rate, thereby extending their shelf life. Priyantono et al. [79] stored shallot bulbs of 3 varieties, namely, Bima Brebes, Tajuk, and Bali Karet , at temperatures of 0°C and 5°C, with RH 65%–70%, and room temperature 25–32°C, with RH 50%–88% for 3 months. The results showed that shallots in the three varieties stored at a temperature of 0°C showed the best quality when compared to other treatments. Cold storage can maintain the freshness and hardness of shallots, because storage at temperatures of 0°C and 5°C can reduce the respiration rate and transpiration rate, so that it can inhibit the metabolic process. Shallots stored at room temperature with low humidity (RH < 65%) will cause the skin to become dry, brittle, cracked, and easily peeled [82].

Most farmers stored shallots with an initial water content of 86.7% for 8 weeks and experienced significant losses of 25.29%. Mutia et al. [29] stored shallots at initial water content conditions of 80% and 85% combined with cold temperatures of 5°C and 10°C (RH 65%–70%). Bima Brebes shallots with an initial water content of 80% and stored at 5°C for 8 weeks had the best quality, with the lowest weight loss of 7.06%, a hardness of 4.38 N, and minimal damage to the bulbs at 0.37%.

### 5.2. CAS

This technique involves controlling the composition of the atmosphere around the product by changing the levels of oxygen (O_2_), carbon dioxide (CO_2_), nitrogen, ethylene, and humidity. This technique has not been widely used because it requires a large amount of funds. From the results of studies conducted on several horticultural products, including shallots, this technology has been proven to extend the shelf life of the best horticultural commodities. Fresh shallots can be stored for more than 6 months with losses of less than 5%.

Bajer and Gajewski [80] reported that storing three shallot cultivars (namely, Conservor, Prisma, and Bonilla) using different atmospheric compositions affected the quality of shallot bulbs for 7 months of storage (typical atmosphere, 5% CO_2_ + 5% O_2_, 5% CO_2_ + 2% O_2_, 2% CO_2_ + 5% O_2_, and 2% CO_2_ + 2% O)_2_. The results showed that CA storage conditions influenced the quality parameters of shallot bulbs, namely, material weight, dry weight, hardness, and dissolved solids content. Under typical storage conditions, the highest weight loss occurred. The dissolved solids content decreased, although not significantly, during storage. At an atmospheric composition of 5% CO_2_ + 5% O_2_, there was a significant increase in dry matter. Dry matter content is the main characteristic of shallot quality.

Chope et al. [81] conducted a study on three types of shallot cultivars (Renate, Ailsa Craig, and SS1), which have different shelf lives (long, medium, and short). These three types of cultivars were stored under CA conditions (3.03 kPa CO_2_; 5.05 kPa O_2_; 2°C). The results showed a significant decrease in abscisic acid content during the first 80 days of storage. Differences in cultivars result in significant variations in several quality parameters, including dry weight, hardness, germination, soluble solids content, fructan concentration, and pyruvic acid concentration. To extend the shelf life of varieties that have a short shelf life, the abscisic acid content can be reduced.

### 5.3. ID

ID can be defined as the process of drying something in a building or structure. This definition encompasses drying with hot circulating air, drying with dehumidification, or simply indoor drying. Drying uses similar equipment, such as the solar dryer used for Persian shallot (*Allium hirtifolium* Boiss), which, being white, cannot be called an in‐store dryer but becomes a solar dryer only [83] or a hybrid solar dryer with LPG gas heating power [84]. An in‐store dryer can also be defined as a field dryer located close to the farmer’s shallot plants on a large enough scale (15 tons) to store bulbs and shallot seeds. The in‐store dryer is also built in the field next to shallot cultivation.

ID can be used to dry shallots into dried whole shallots that are ready for sale and transportation, known in Indonesia by the term “kering askip” [75, 85]. Furthermore, after drying, the dried askip bulb can be stored in the same in‐store dryer [75] or transported further [85]. There are two types of ID, namely, those for lowlands, such as the Brebes model 24 m DPL with an average temperature of 25°C–33°C [56], and those for highlands, such as in Jorong Koto, Nagari Sungai Nanam, Lembah Gumanti District, Solok Regency. The plateau with an altitude of 930 m above sea level has low day and night temperatures and can even reach 19–24°C. The main building of the instore dryer can be categorized into three parts: the product storage area, the heat source, and the air circulation system [75, 85]. All parts are operated manually to achieve the desired specific temperature and humidity [75]. The main building of ID can be constructed from various materials, such as red brick walls with fiberglass roofs [56] or light steel frame walls with fiberglass roofs [85].

Part of the heat source is to create hot air that has low RH. There are two types of heat sources, wood‐fired stoves and large blowers that blow low RH air into the room [75, 76]. The other type is a husk‐powered energy source equipped with a diesel engine to blow hot air. For the ID type, hot air is also blown from the other end [85].

The air circulation part can utilize various types of fans, including centrifugal‐type electric fans, coaxial‐type electric fans, and roof wind‐driven ventilator fans [75, 76, 85]. The coaxial type has greater suction power than the centrifuge. Both types of fans can only be installed on the side wall. A fan with wind power can be more energy efficient, but it can only rotate when wind is blowing through it. Wind power fans come in various sizes to meet our needs. In addition to commercial fans, windows can also be installed on the ID wall, allowing them to be opened/closed manually to reduce hot or humid air. To add dry air, one can burn wood in the furnace without a blower [75]; alternatively, a furnace that heats the air and then blows it using a large‐capacity blower can be used [76]. A husk‐fired furnace can also serve as a heat source during nighttime, with assistance from a diesel engine powering the blower fan [85].

## 6. Postharvest Technology Challenge and Future Prospects for Shallots

The enormous demand for shallots, especially in Indonesia, is one of the excellent prospects for the future, especially to maintain the supply of fresh shallots available at all times. The use of postharvest technology in the field is essential. The use of ID for curing and drying will greatly help shallot farmers to avoid damage to fresh shallots, especially during the rainy season. The use of ID, in addition to reducing damage to shallots due to faster curing and drying times, can also serve as a storage space. The temperature and humidity in ID are more controlled than when dried shallots are stored in an open space or on the warehouse floor. In ID, there are storage shelves that can protect fresh shallots from contaminants or excessive humidity. Another technology that can also be applied is storage with CAS. CAS can maintain the freshness of shallots for more than 6 months with losses of less than 5%. Although this technology requires a relatively high initial investment, it can reduce storage costs when used in large quantities.

To prevent losses due to postharvest damage from being severe, microbial damage control treatments are needed. Control of microbiological damage to shallots in the future is expected to use natural ingredients that are safe for human health and the environment. Some essential oils and plant extracts contain antimicrobial active ingredients. The selection of antimicrobials based on essential oils and natural extracts is safer for health and environmentally friendly. Residues in food resulting from the use of pesticides and toxic chemicals are harmful to consumers [86]. Since the cause of shallot rot is mostly fungi, microbial control is focused on fungi. Shen [87] reported that cinnamaldehyde, an essential oil from cinnamon, has high antifungal activity, to maintain the quality of fresh horticultural products during storage. Cinnamaldehyde in the form of 0.3 g/L vapor in the air can control *F. solani* attacks on potatoes during storage [88]. An essential oil, citronella oil is effective in inhibiting the growth of pathogenic fungus *Alternaria porri*, the causative agent of purple spot disease in leek onion plants (*Allium fistulosum* L.), at concentrations of 3% and 4% [89]. Extracts of Ethiopian mustard (*Brassica carinata*) and rapeseed (*B. napus*) showed high inhibition of the growth of the shallot *F. oxysporum* f. sp. *cepae* pathogen [20]. Application of nano‐chitosan at 100 ppm + nano‐silica at 100 ppm was effective in suppressing the development of shallot twisting disease of 56.3%, comparable to mankozeb which was 50.5% [90].

Smoking with the external pyrolysis method can reduce the infection of pathogenic fungi in shallot bulbs and extend the dormancy period [65]. Smoking treatment with the pyrolysis method does not affect the physiological quality of shallot bulb seeds (germination power and maximum growth potential). Liquid smoke from coconut shell pyrolysis contains acetic acid components of around 51.6% at 200°C combustion, 50.88% at 250°C, and 39.98% at 300°C. Increasing the temperature of the pyrolysis process increases the number of components. The number of components in liquid smoke at a pyrolysis temperature of 200°C–300°C is 57 [91]. Hadanu and Apituley [92] found that the volatile components of coconut shell liquid smoke were 90.75% phenol derivatives, 3.73% trimethoxybenzene derivatives, 3.71% 2‐cyclopenten‐1‐one derivatives, and 1.81% 2‐furanmethanol compounds, and phenol derivatives were the main components of the liquid smoke.

## 7. Conclusions

Efforts to maintain the quality of shallots through effective postharvest handling are crucial for reducing losses, maintaining quality, and enhancing market value. Combining improved harvesting methods, drying techniques, storage solutions, and farmer education can significantly extend shelf life and profitability. Proper postharvest handling also maintains the nutritional and phytochemical composition of the product. Continuous research, investment, and dissemination of these practices are essential for the future growth of the shallot industry.

## Funding

No funding was received for this manuscript.

## Conflicts of Interest

The authors declare no conflicts of interest.

## Data Availability

The data that support the findings of this study are available from the corresponding author upon reasonable request.
